# Supplementary material for: Robust proteome profiling of cysteine-reactive fragments using label-free chemoproteomics
Source: Nat Commun. 2025 Jan 2;16:73. doi: 10.1038/s41467-024-55057-5 (PMC11697256; doi:10.1038/s41467-024-55057-5)
Supplement: Supplementary file 1 — Supplementary Information [file 41467_2024_55057_MOESM1_ESM.pdf]

Supplementary Information

Robust proteome profiling of cysteine-reactive fragments using label-free chemoproteomics

George S. Biggs†,[1,2,3] Emma E. Cawood†,[1,4] Aini Vuorinen,[1,2,3] William J. McCarthy,[2] Harry Wilders,[1,5] Ioannis G. Riziotis,[1,6] Antonie J. van der Zouwen,[2] Jonathan Pettinger,[1] Luke Nightingale,[6] Peiling Chen,[7] Andrew J. Powell,[1] David House,[1] Simon J. Boulton,[4] J. Mark Skehel,[3] Katrin Rittinger*,[2] Jacob T. Bush*[1]

[1] Crick-GSK Biomedical LinkLabs, GSK, Gunnels Wood Road, Stevenage, Hertfordshire, SG1 2NY, UK.

[2] Molecular Structure of Cell Signalling Laboratory, The Francis Crick Institute, 1 Midland Road, London, NW1 1AT, UK.

[3] Proteomics Science Technology Platform, The Francis Crick Institute, 1 Midland Road, London, NW1 1AT, UK.

[4] DSB Repair Metabolism Laboratory, The Francis Crick Institute, 1 Midland Road, London NW1 1AT, UK.

[5] University of Strathclyde, Pure and Applied Chemistry, 295 Cathedral Street, Glasgow G1 1XL, UK.

[6] Software Engineering and AI, The Francis Crick Institute, 1 Midland Road, London NW1 1AT, UK.

[7] GSK Chemical Biology, GSK, 1250 S Collegeville Rd, Collegeville, PA 19426, USA.

† These authors contributed equally to this work.

* Joint corresponding authors.

Contents

SUPPLEMENTARY FIGURES **3**

Supplementary Figure 1. Data depth and completeness........ 3

Supplementary Figure 2. Overlap of peptide, cysteine, and protein detection in HEK293T and Jurkat lysates........ 4

Supplementary Figure 3. Estimating the size of the cysteinome that is ‘feasibly-detectable’ using IA-DTB chemoproteomics. 4

Supplementary Figure 4. Surface exposure of residues detected through the IA-DTB probe in HEK293T lysate. 5

Supplementary Figure 5. Chemical and physical properties of our 80-member library of chloroacetamide-functionalised fragments. 6

Supplementary Figure 6. Data from the 80-compound screen performed in HEK293T and Jurkat lysate. 8

Supplementary Figure 7. Concentration-response curves for all peptides with concentration-dependent interactions. 9

Supplementary Figure 8. Concentration-response curves for all detected cysteine residues in TPMT, VCP, MOB4, and MKLN1 with the hit fragments for these proteins. 11

Supplementary Figure 9. MOB4, NIT1, and NIT2 concentration-response data for a panel of compounds derived from PP48. 12

SUPPLEMENTARY TABLES **13**

Supplementary Table 1. SMILES for all chloroacetamides used in this study........ 13

Supplementary Table 2. Number of unique peptides detected across all experiments, before and after data filtering. 15

Supplementary Table 3. The diaPASEF method for chemoproteomics data acquisition on a Bruker timsTOF Pro 2. 16

Supplementary Table 4. The diaPASEF method for global proteomics data acquisition on a Bruker timsTOF Pro 2. 17

SUPPLEMENTARY REFERENCES **18**

Supplementary Figures

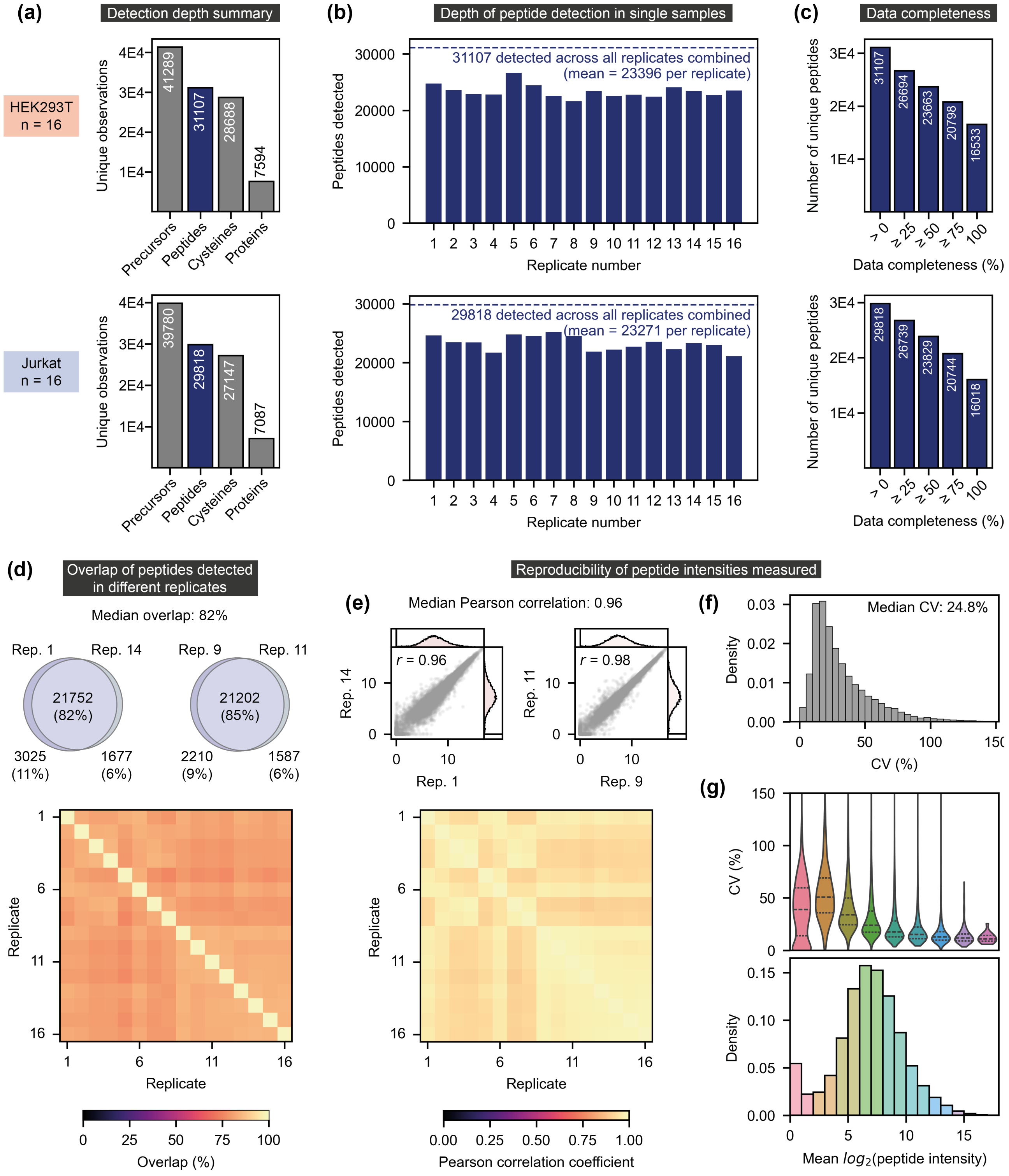

Supplementary Figure 1. Data depth and completeness. The detection depth and completeness of chemoproteomics data from HEK293T and Jurkat cell lysates. Data was obtained using 16 replicate DMSO control samples prepared from each lysate, and is summarised based on the total detection depth across all replicates **(a)**, the number of peptides detected in each individual replicate sample **(b)**, and the number of peptides detected at varying levels of data completeness **(c)**. In **(d)**-**(e)**, a more detailed view of peptide detection reproducibility is shown for the HEK293T lysate data, showing both the overlap in the peptides detected by each replicate (median overlap of 82% between any two replicates) and the correlation between the (log_2-transformed) intensity of these peptides (median Pearson correlation between any two replicates of 0.96). Heatmaps summarise the pairwise similarity between replicates, and Venn diagrams of detected peptides **(d)** and correlation plots of log_2-transformed peptide intensities **(e)** are shown for representative replicates at the top of both figures.

The coefficient of variation (CV) distribution of the detected peptides shows that the vast majority of peptides are detected with good signal-to-noise **(f)**, and that those peptides with high CV values are associated with low peptide intensities **(g)**. For both **(f)** and **(g)**, n=28,954 (the number of peptides detected in at least 2 replicates). In the violin plots in **(g)**, the dotted lines indicate the quartiles of the data.

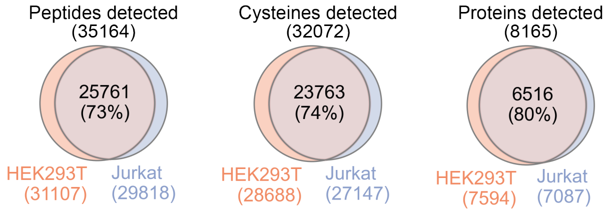

Supplementary Figure 2. Overlap of peptide, cysteine, and protein detection in HEK293T and Jurkat lysates. Data was obtained using 16 replicate DMSO control samples from each lysate.

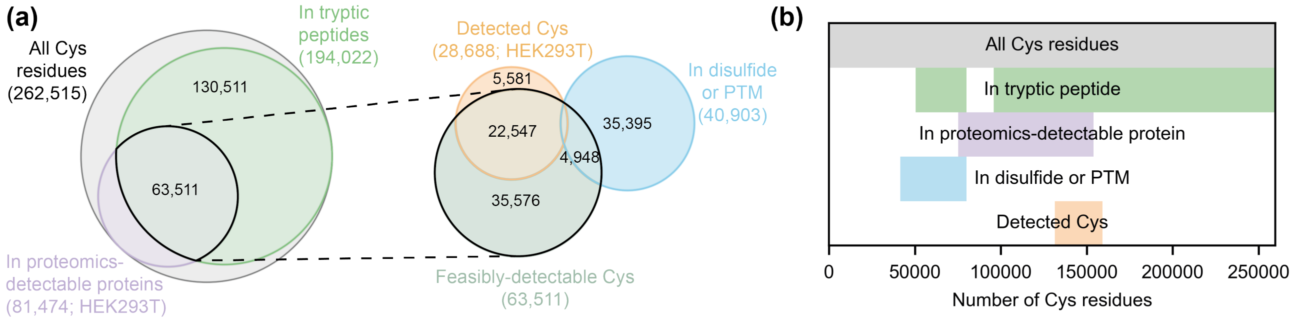

Supplementary Figure 3. Estimating the size of the cysteinome that is 'feasibly-detectable' using IA-DTB chemoproteomics. Data is summarised both through Venn diagrams **(a)** and a supervenn plot **(b)**.[1] Cysteine residues that are detected through IA-DTB chemoproteomics must have fulfilled two criteria: they were present within tryptic peptides of a suitable size for detection by mass spectrometry and they were present in proteins that are sufficiently abundant in the cell lysate. We have performed an *in silico* trypsin digestion of all proteins (~20,000) in the human proteome and estimate that, of the ~263,000 unique cysteine residues (grey), ~194,000 of these lie within tryptic peptides of an appropriate size (see experimental methods) (green). We have approximated the number of cysteines present in proteins of sufficient abundance in HEK293T lysate by assessing which proteins can be detected by standard proteomics methods, which accounted for ~81,000 cysteines (purple). Taken together, this gives an estimate of ~64,000 cysteine residues that lie within tryptic sequences of sufficiently abundant proteins in HEK293T lysate. Excluding the ~40,000 cysteine residues that are annotated in UniProt as being involved in disulfide bonds or other post-translational modifications (blue), our IA-DTB chemoproteomics experiments performed in HEK293T lysate (n=16 DMSO control samples) detect ~40% of the 'feasibly-detectable' cysteine residues (orange).

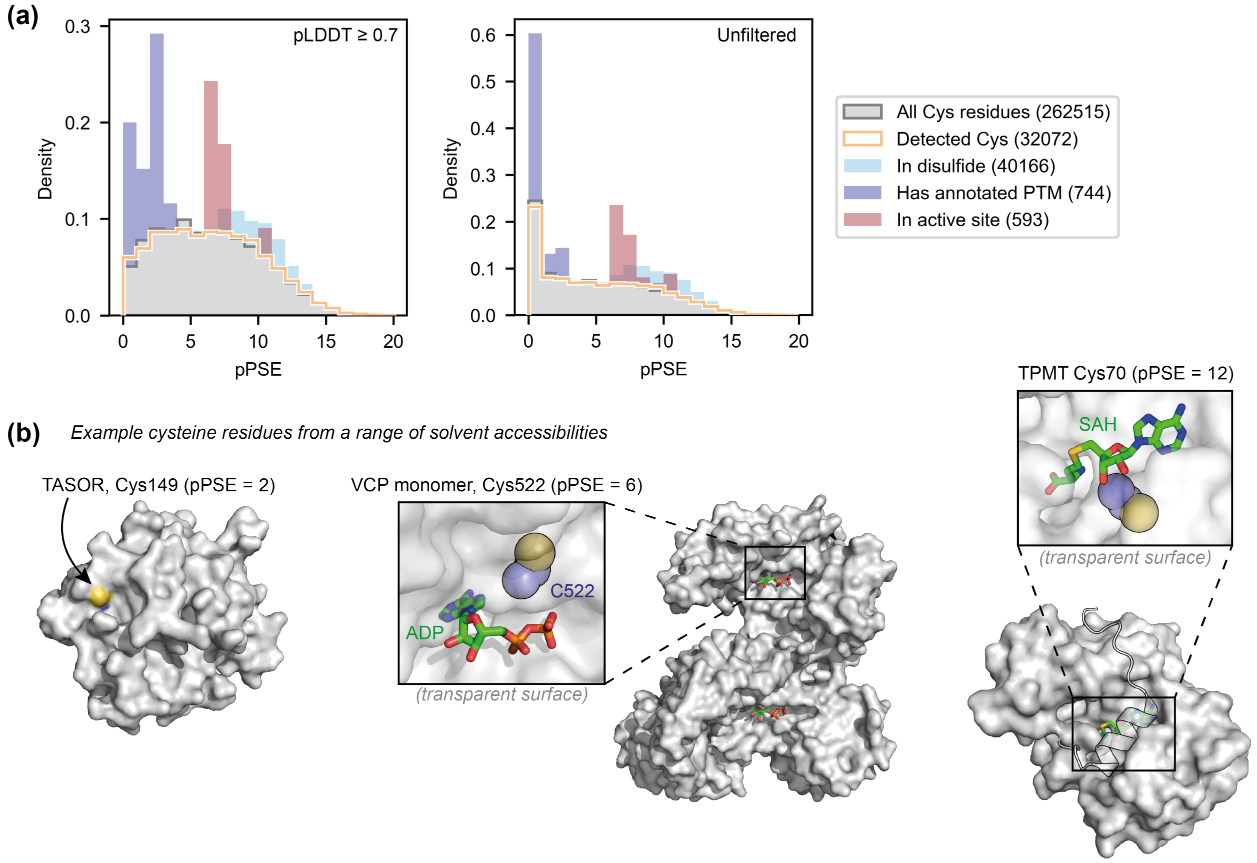

Supplementary Figure 4. Surface exposure of residues detected through the IA-DTB probe in HEK293T lysate. We detect cysteine residues from a range of solvent accessibilities (quantified using the pPSE metric) (as shown in the histogram in **(a)**),[2] including fully solvent-exposed residues (*e.g.*, TASOR Cys149, pPSE 2; PDB 6TL1),[3] residues in metabolite binding pockets (*e.g.*, VCP Cys522, pPSE 6; PDB 5FTJ),[4] and residues that lie deep within binding channels (*e.g.*, TPMT Cys70, pPSE 12; PDB 2H11; cartoon representation used for residues 17-39) **(b)**.[5] While pPSE values were calculated using AlphaFold2 models, the protein structures shown are from experimentally validated structures.[6]

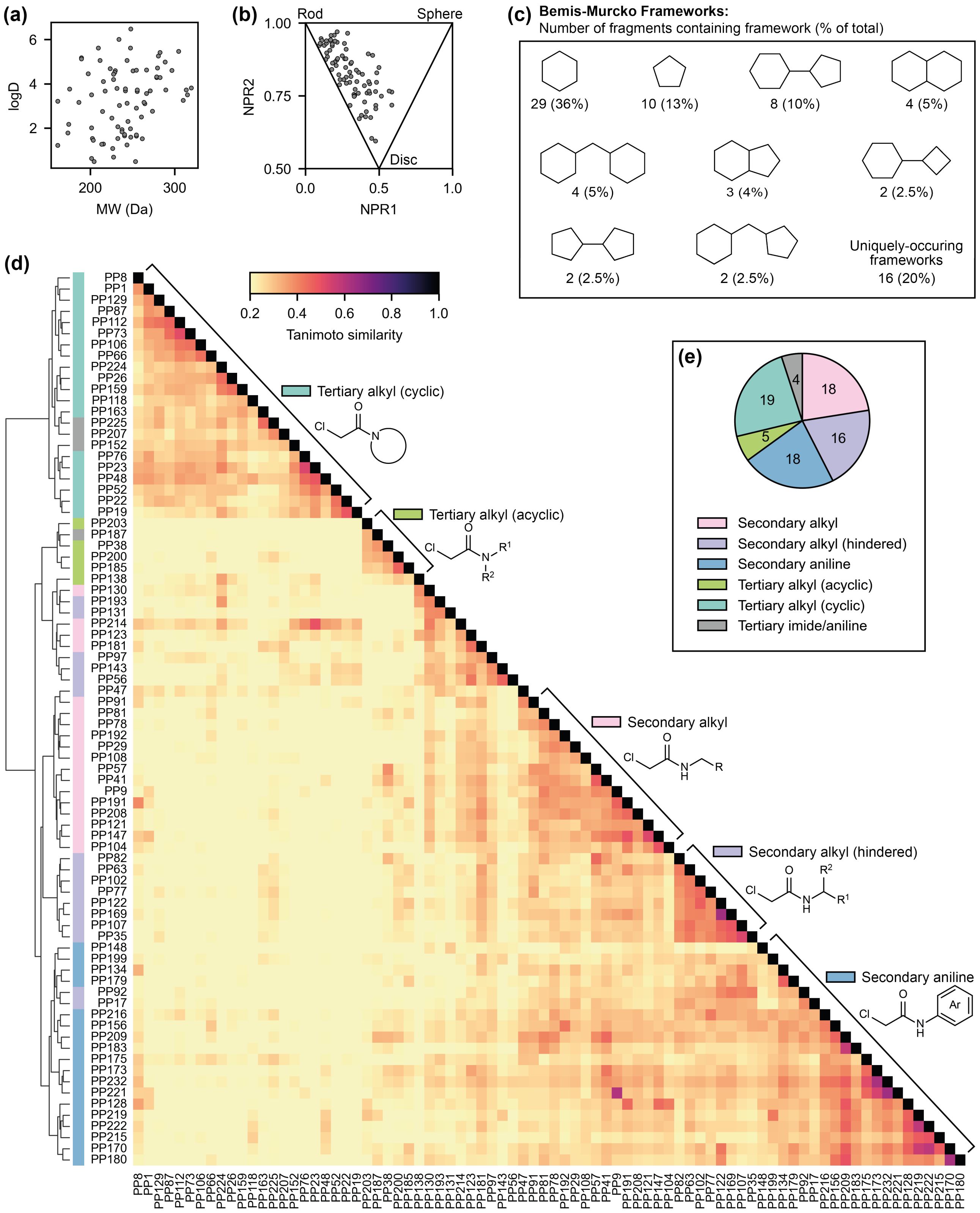

Supplementary Figure 5. Chemical and physical properties of our 80-member library of chloroacetamide-functionalised fragments. (a) All compounds possess a molecular weight that is consistent with the size range typically recommended for small molecule fragments.[7] Compound lipophilicity is represented as a log_{10}-transformed distribution coefficient (logD). **(b)** The library as a whole shows a diversity of physical shapes, based on the normalised principal moment of inertia ratios (NPR) obtained for these fragments from their predicted three-dimensional structures.[8] **(c)** As the size limit of small molecule fragments places restraints on the types of molecular frameworks that can be explored, the vast majority of compounds in our library consist of a small number of frameworks. However, the vast majority of the scaffolds in our library have previously been identified as structures that are well-suited to binding proteins.[9] **(d)** Tanimoto similarity matrix, based on Morgan molecular

fingerprints, with hierarchical clustering. In general, there is little similarity between library members, and the clusters of similarity that are detected are largely driven by structural features adjacent to the chloroacetamide functional group. **(e)** Pie chart illustrating the distribution of structural features adjacent to the chloroacetamide functional group.

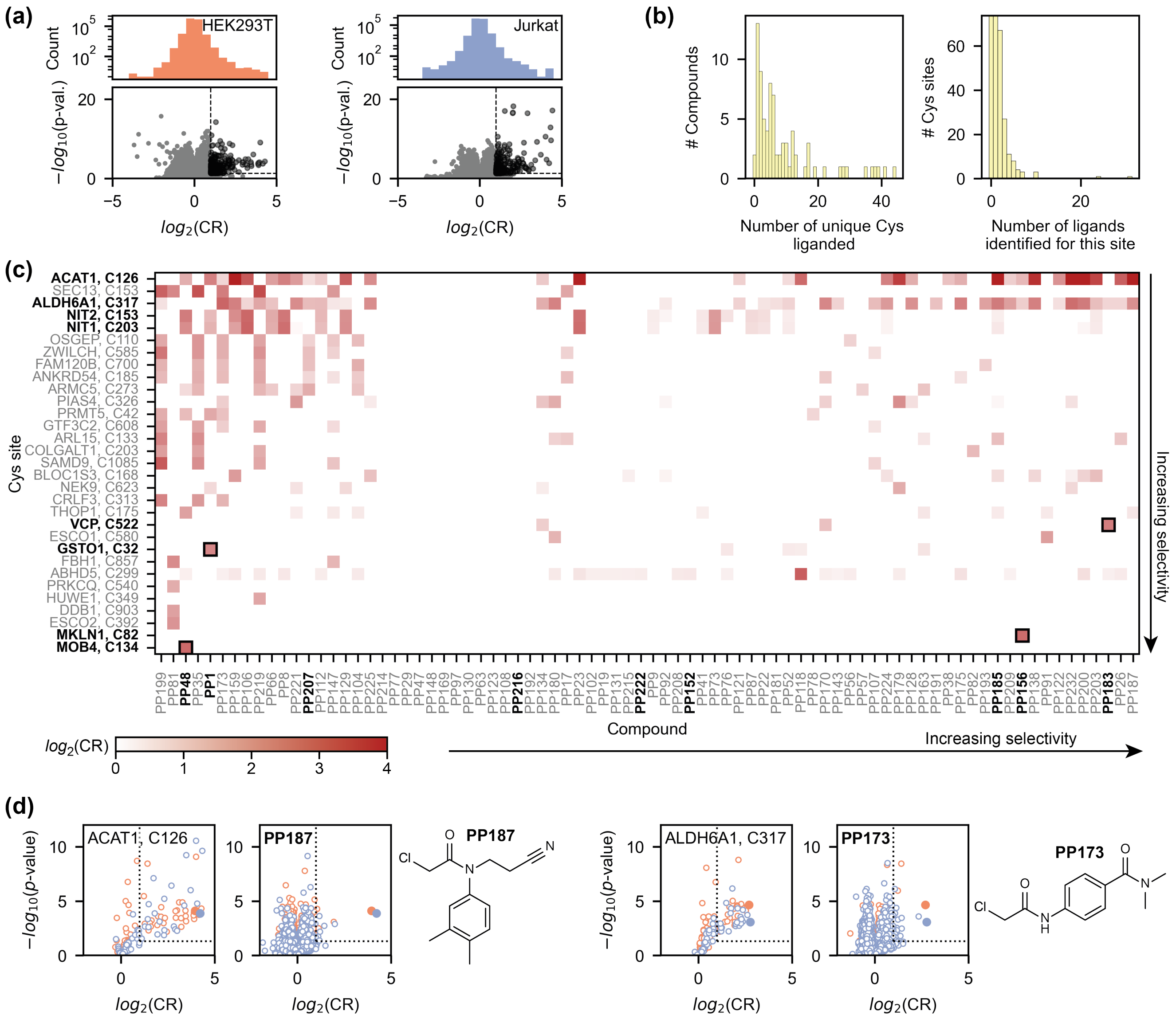

Supplementary Figure 6. Data from the 80-compound screen performed in HEK293T and Jurkat lysate. (a) Volcano plot and log_2(CR) distributions of all the data obtained in HEK293T (left) and Jurkat (right) lysate (n=4 for compound-treated samples, n=16 for DMSO control samples). Each data point reports on the binding of an individual compound (50 µM) to an individual cysteine-containing peptide. **(b)** Distribution of compound (left) and cysteine (right) promiscuity. **(c)** Heatmap of all the interactions detected in Jurkat lysate. For clarity, this heatmap only includes cysteines that are liganded by at least compound with log_2(CR) ≥ 1.5. Selective interactions are highlighted by black boxes, and the compounds/Cys sites that are discussed in the main text are labelled in bold. **(d)** Volcano plots for two active site cysteine residues (ACAT1 Cys126 and ALDH6A1 Cys317). While these cysteine residues bind many compounds, two compounds were identified that only bind these cysteine sites (PP187 and PP173, respectively). HEK293T data: orange; Jurkat data: blue. All p-values were calculated using Welch's t-test (two-sided).

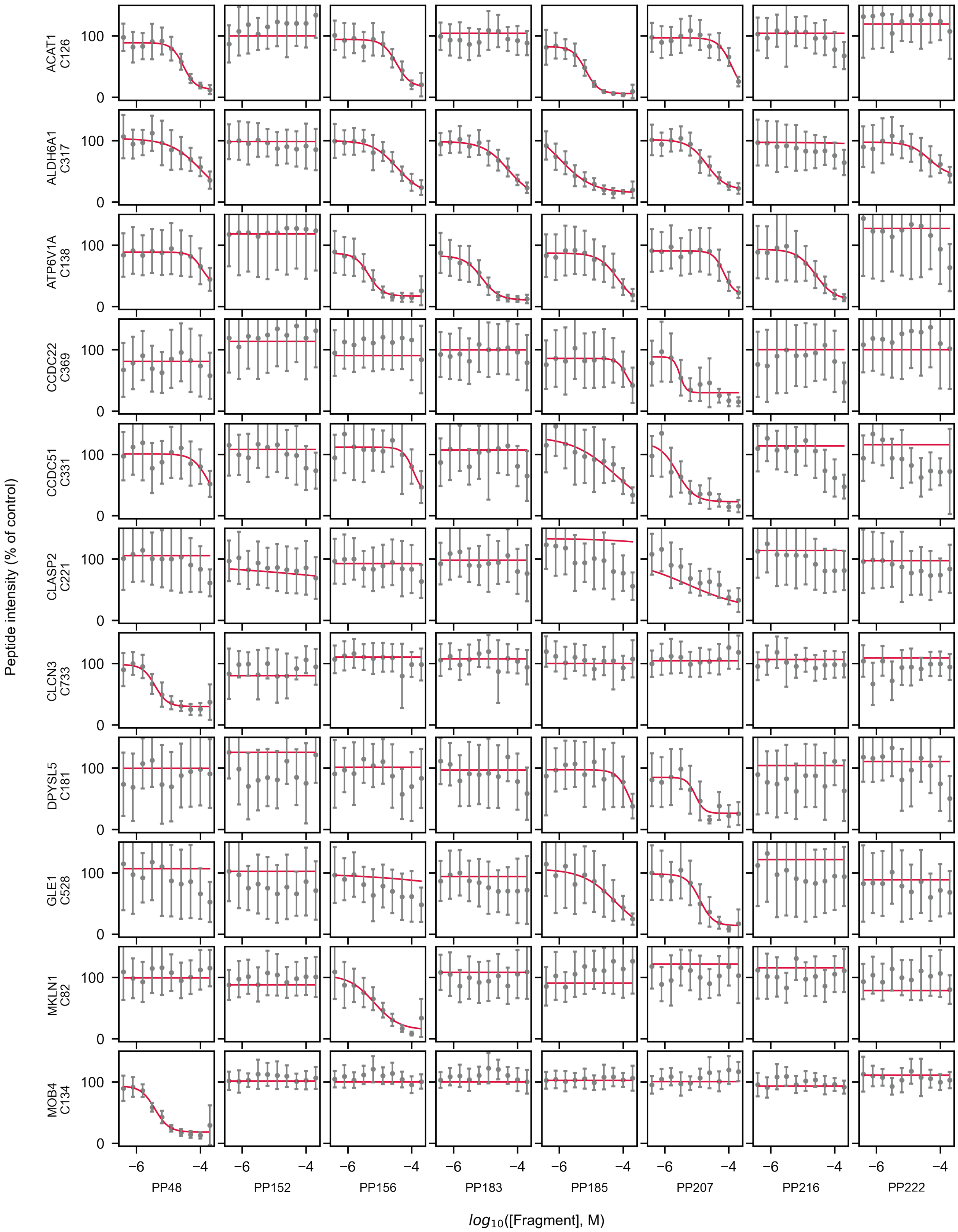

Supplementary Figure 7. Concentration-response curves for all peptides with concentration-dependent interactions. Continued over the page.

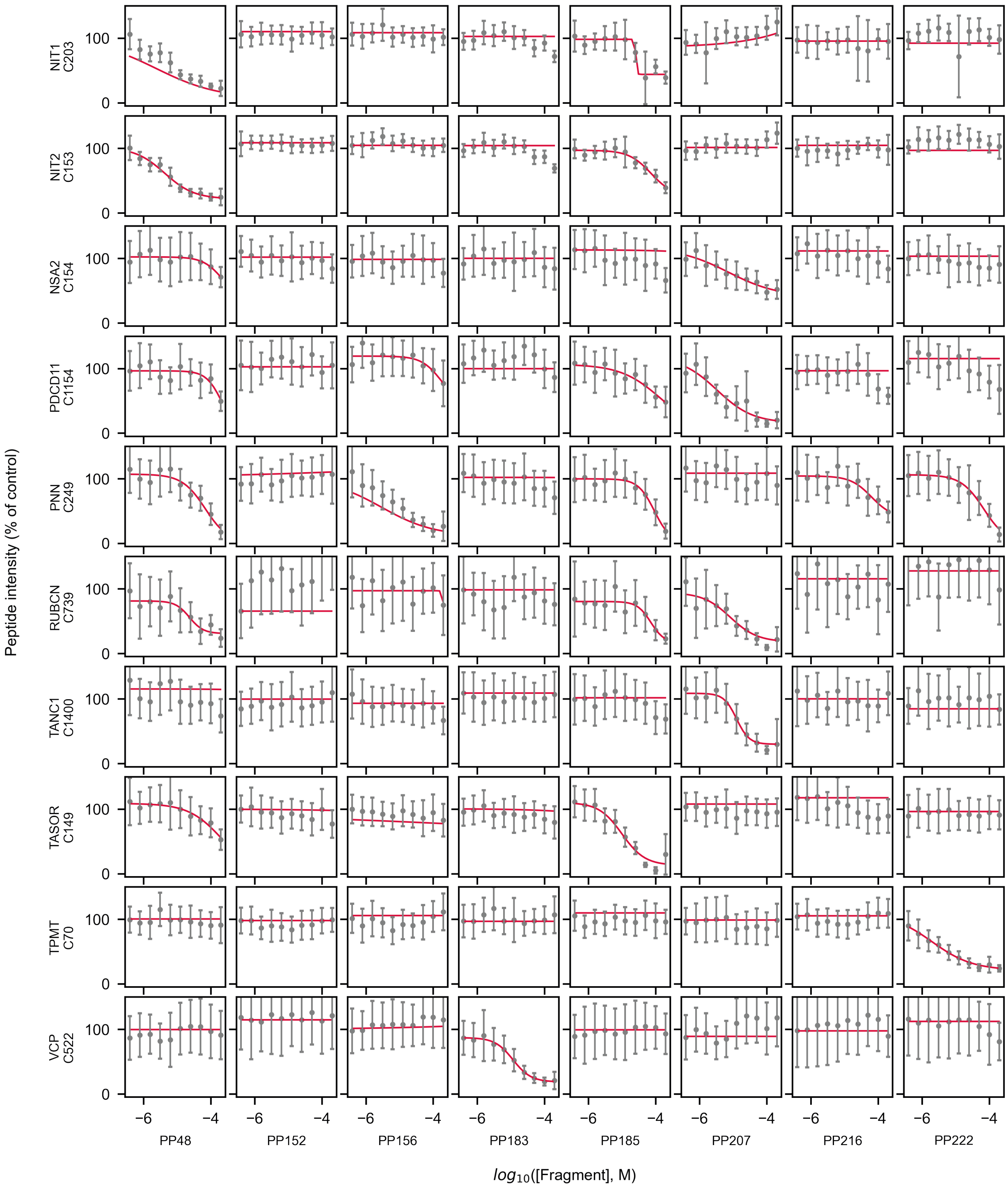

Supplementary Figure 7. Concentration-response curves for all peptides with concentration-dependent interactions. Data points represent the mean peptide intensity (relative to the DMSO control; error bars represent the standard deviation) measured at varying concentration of eight different chloroacetamides. Red lines represent the results of fitting a four-parameter logistic function to the data, which was used to identify concentration-dependent interactions. The resulting pTE_{50} values are summarised by the heatmap in Fig. 4e. Compound treatments were performed in HEK293T lysate (n=4 for compound-treated samples, n=25 for DMSO control samples).

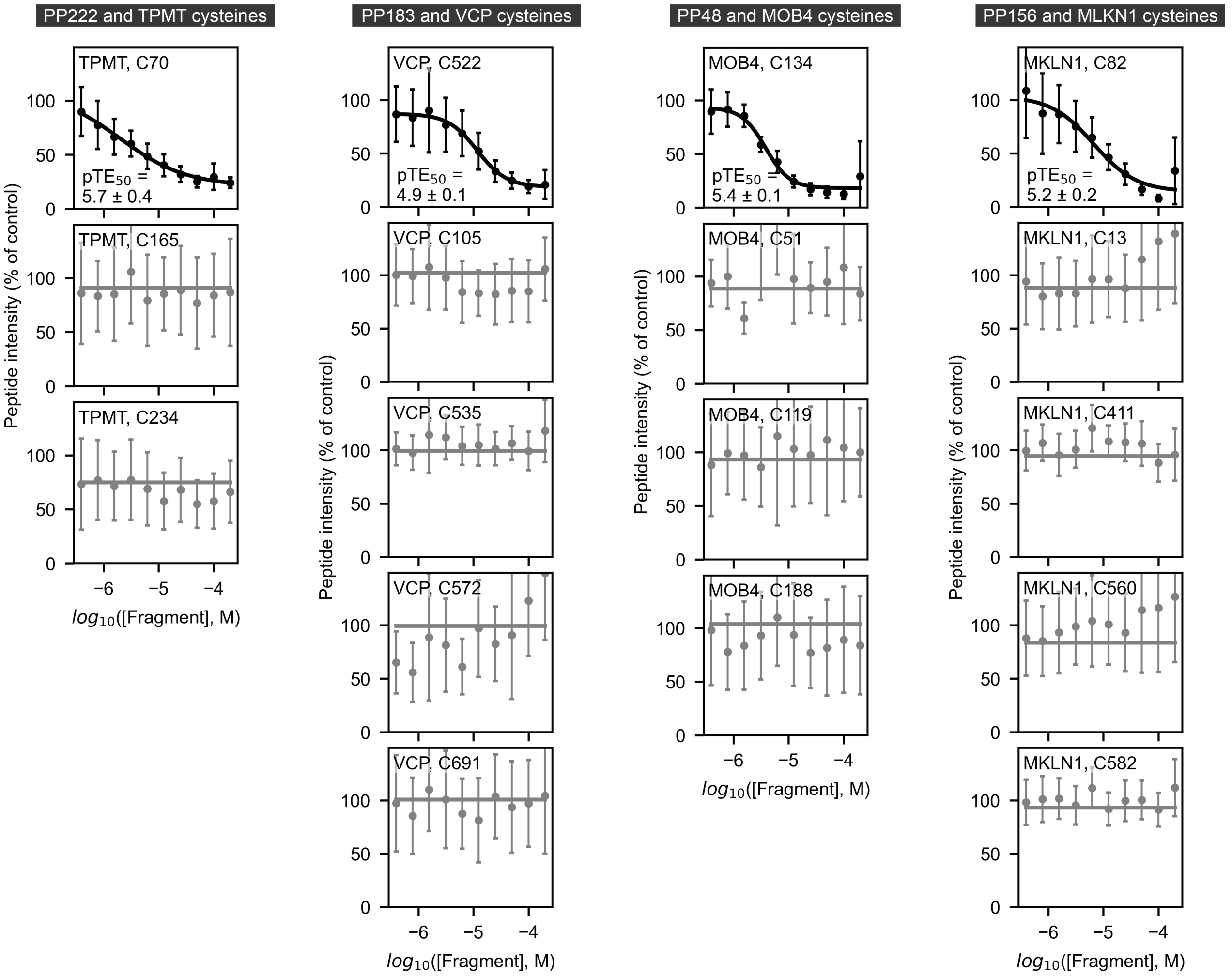

Supplementary Figure 8. Concentration-response curves for all detected cysteine residues in TPMT, VCP, MOB4, and MKLN1 with the hit fragments for these proteins. Data points and error bars represent the mean peptide intensity and standard deviation, respectively. Compound treatments were performed in HEK293T lysate (n=4 for compound-treated samples, n=25 for DMSO control samples).

(a)

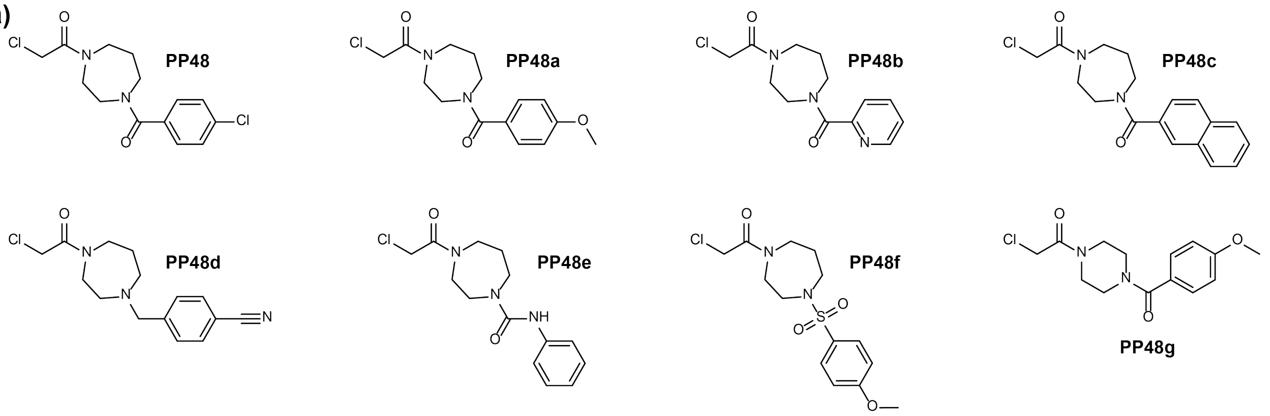

(b)

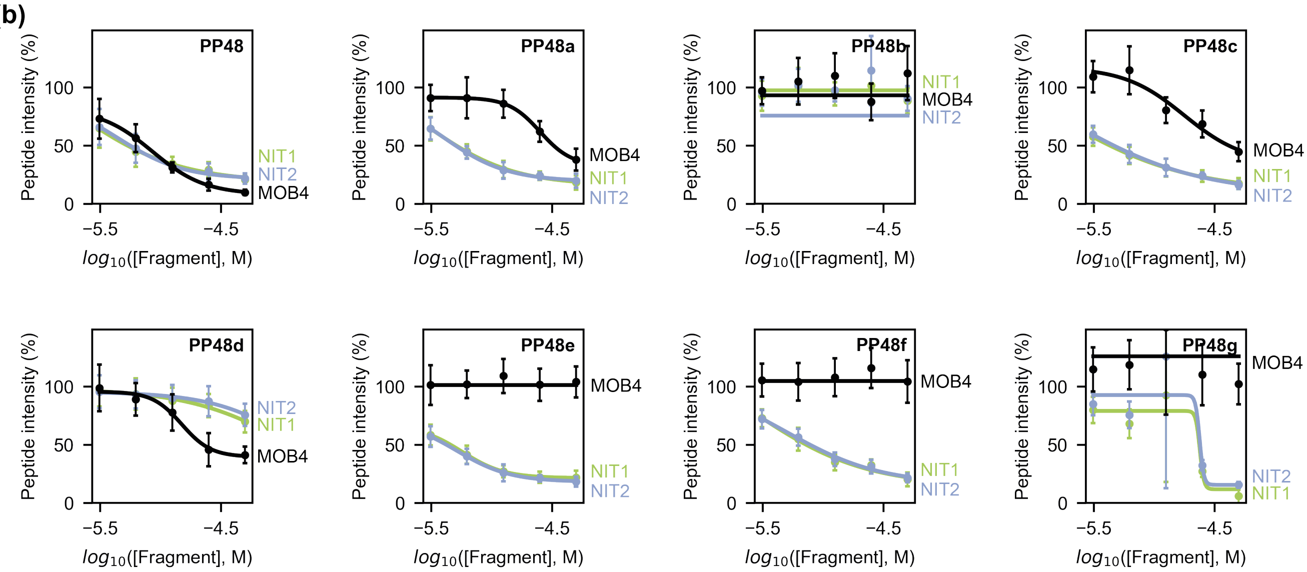

Cys target	PP48	PP48a	PP48b	PP48c	PP48d	PP48e	PP48f	PP48g
MOB4 Cys134	5.1 ± 0.1	4.6 ± 0.1	NB	4.8 ± 0.1	4.8 ± 0.1	NB	NB	NB
NIT1 Cys203	5.7 ± NE	5.5 ± 0.9	NB	5.8 ± NE	3.9 ± NE	5.2 ± 0.1	5.6 ± NE	4.6 ± NE
NIT2 Cys153	5.4 ± 0.6	5.4 ± 0.4	NB	5.8 ± NE	3.8 ± NE	5.3 ± 0.2	5.3 ± 0.4	4.6 ± NE

(c)

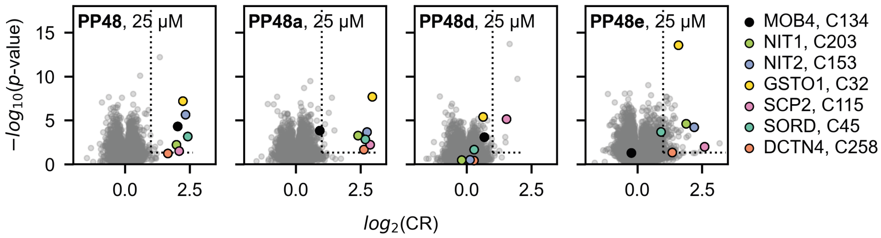

Supplementary Figure 9. MOB4, NIT1, and NIT2 concentration-response data for a panel of compounds derived from PP48. (a) Structures of the seven analogues of PP48 that were used to explore SAR around binding to MOB4, NIT1, and NIT2. **(b)** Concentration-response data are shown as the mean ± standard deviation for each compound concentration alongside the logistic regression curve. The pTE_{50} values and associated errors from this regression analysis are shown in the table. Compound treatments were performed in HEK293T lysate (n=4 for compound-treated samples, n=32 for DMSO control samples). NB: no binding detected. NE ('not estimated') refers to errors whose value could not be accurately estimated. **(c)** Volcano plots displaying data acquired in live HEK293T cells (25 μM compound treatment, 2-hour incubation; n=3 for compound-treated samples; n=12 for DMSO control samples) for PP48 compared to three SAR analogues. Data is the same as shown in Figure 5c. All *p*-values were calculated using Welch's *t*-test (two-sided).

Supplementary Tables

Supplementary Table 1. SMILES for all chloroacetamides used in this study.

Compound number	SMILES	Enamine Catalogue ID
PP1	Fc1ccc(cc1)S(=O)(=O)C1CCN(CC1)C(=O)CCl	Z3433759316
PP8	Fc1cccc(NC(=O)CN2CCN(CC2)C(=O)CCl)c1	Z147647796
PP9	CS(=O)(=O)c1ccc(CNC(=O)CCl)cc1	Z561690358
PP17	CC(Cc1cc(C)[nH]n1)NC(=O)CCl	Z1562149874
PP19	ClCC(=O)N1CCCCC11CCOCC1	Z3072692879
PP22	ClCC(=O)N1CC2(C1)CCOCC2	Z3217399684
PP23	ClCC(=O)N1CCN(CC1)C(=O)N1CCCCC1	Z258606556
PP26	ClCC(=O)N1CCCCCC1c1ncon1	Z3766261126
PP29	CCc1c[nH]c(CNC(=O)CCl)n1	Z2855088493
PP35	CC(NC(=O)CCl)c1noc(Cc2ccccc2)n1	Z1562147278
PP38	Cc1ccc(CN(CCO)C(=O)CCl)cc1	Z3766270238
PP41	Cc1ccc(cc1)C(=O)NCCNC(=O)CCl	Z644776932
PP47	Cc1cccc(c1)N1CCC(NC(=O)CCl)C1=O	Z3217399408
PP48	ClCC(=O)N1CCCN(CC1)C(=O)c1ccc(Cl)cc1	Z1562129258
PP52	ClCC(=O)N1CCOC2(CCC2)CC1	Z3073542106
PP56	CNC(=O)C1(CCCCC1)NC(=O)CCl	Z567138138
PP57	Cc1ccc(CC(CO)CNC(=O)CCl)cc1	Z3766270656
PP63	NC(=O)C(Cc1ccccc1)NC(=O)CCl	Z1562130066
PP66	Cc1conc1C1CN(C1)C(=O)CCl	Z3766268825
PP73	ClCC(=O)N1CCCC(C1)c1ncc[nH]1	Z1562136602
PP76	CS(=O)(=O)N1CCCN(CC1)C(=O)CCl	Z1562122122
PP77	Cn1nccc1C(NC(=O)CCl)c1ccccc1	Z3766264674
PP78	Cc1ccn2cc(CNC(=O)CCl)nc2c1	Z1562128734
PP81	Cc1ccc(C)c(Oc2ccc(CNC(=O)CCl)cn2)c1	Z561892770
PP82	Cc1ccc(CC(NC(=O)CCl)c2ccccn2)cc1	Z1562142058
PP87	ClCC(=O)N1CCC(Cc2nccs2)C1	Z2981785917
PP91	Cc1cccc(c1)C(CNC(=O)CCl)N1CCOCC1	Z562028592
PP92	CC(NC(=O)CCl)c1ccc2[nH]ncc2c1	Z3766268912
PP97	ClCC(=O)NC1(CCC1)c1ncccn1	Z2863946069
PP102	COCC[C@@H](NC(=O)CCl)c1ccccc1	Z4527731482
PP104	Fc1cnc(CCNC(=O)CCl)c(F)c1	Z3217401712
PP106	ClCC(=O)N1CC(C1)c1ccc(Br)cn1	Z4527731370
PP107	CC(NC(=O)CCl)c1csc(n1)-c1ccccc1	Z3766259965
PP108	Oc1nc2CCCCc2cc1CNC(=O)CCl	Z2763293066
PP112	ClCC(=O)N1CCC(C1)c1ccn[nH]1	Z3766265762
PP118	ClCC(=O)N1CCc2nc[nH]c2C1c1ccc(Cl)cc1	Z3766262081
PP121	ClCC(=O)NCCc1ccc2OCCOc2c1	Z147648024
PP122	COc1ccccc1C(C)NC(=O)CCl	Z85921033
PP123	CN(C)C1(CNC(=O)CCl)CCCCC1	Z57044257
PP128	Fc1ccc(NC(=O)CCl)c(F)c1	Z56896319
PP129	ClCC(=O)N1CCC(CC1)c1nc2ccccc2o1	Z57980312

PP130	ClCC(=O)NCC1CCC1	Z411906316
PP131	FC(F)(F)C1CCC(CC1)NC(=O)CCl	Z258588862
PP134	Cc1nc(cs1)-c1cccc(NC(=O)CCl)c1	Z57984668
PP138	CN(C1CCCCC1)C(=O)CCl	Z56891381
PP143	ClCC(=O)NC1(CCCC1)C#N	Z56346983
PP147	Fc1ccc(CCNC(=O)CCl)cc1	Z56822826
PP148	FC(F)(F)Cc1nnc(NC(=O)CCl)o1	Z1021388618
PP152	ClCC(=O)N1CCCC1=O	Z56900890
PP156	Cc1cc(NC(=O)CCl)no1	Z56883164
PP159	ClCC(=O)N1CCCC1c1ccsc1	Z561606520
PP163	OC(=O)C1Cc2ccccc2CN1C(=O)CCl	Z56922126
PP169	CC(NC(=O)CCl)c1cccc2ccccc12	Z147651784
PP170	COc1ccc(Cl)cc1NC(=O)CCl	Z56896294
PP173	CN(C)C(=O)c1ccc(NC(=O)CCl)cc1	Z56979396
PP175	CC1CN(CC(C)O1)c1ccc(NC(=O)CCl)cc1	Z945125718
PP179	COc1ccc(cc1)-c1csc(NC(=O)CCl)n1	Z56887787
PP180	COc1cc(Cl)c(C)cc1NC(=O)CCl	Z56896263
PP181	ClCC(=O)NCC1(CC1)c1ccccc1	Z258588668
PP183	Cc1nnc([nH]1)-c1ccc(C)cc1NC(=O)CCl	Z1416063849
PP185	COc1cccc(CN(C)C(=O)CCl)c1	Z57057722
PP187	Cc1ccc(cc1C)N(CCC#N)C(=O)CCl	Z199424084
PP191	Fc1cccc(CNC(=O)CCl)c1	Z362629546
PP192	Cc1noc(C)c1CNC(=O)CCl	Z1138459452
PP193	CC1CCCCC1NC(=O)CCl	Z56853743
PP199	ClCC(=O)Nc1c(cnn1-c1ccccc1)C#N	Z353720732
PP200	CN(Cc1ccccc1C)C(=O)CCl	Z57051069
PP203	CCN(Cc1ccc(cc1F)C#N)C(=O)CCl	Z561826190
PP207	ClCC(=O)N1c2ccccc2NC(=O)C11CCCC1	Z169854330
PP208	ClCC(=O)NCc1ccco1	Z56891404
PP209	Cc1ccc(C)c(NC(=O)CCl)c1	Z56867229
PP214	ClCC(=O)NCC(=O)N1CCCC1	Z258588558
PP215	ClCC(=O)Nc1cc(Cl)ccc1-n1cncn1	Z57040453
PP216	CC(C)n1nccc1NC(=O)CCl	Z56347133
PP219	ClCC(=O)Nc1cc(Cl)ccc1C#N	Z56889402
PP221	CS(=O)(=O)c1ccc(NC(=O)CCl)cc1	Z199423514
PP222	OC(=O)c1ccc(Cl)cc1NC(=O)CCl	Z56910423
PP224	CC1CCCCN1C(=O)CCl	Z56853744
PP225	CC1Cc2ccccc2N1C(=O)CCl	Z56869453
PP232	CC(=O)Nc1ccc(NC(=O)CCl)cc1	Z56823002
PP48a	COc1ccc(cc1)C(=O)N1CCCN(CC1)C(=O)CCl	Z1562144566
PP48b	ClCC(=O)N1CCCN(CC1)C(=O)c1ccccn1	Z1562129214
PP48c	ClCC(=O)N1CCCN(CC1)C(=O)c1ccc2ccccc2c1	Z5775137836
PP48d	ClCC(=O)N1CCCN(Cc2ccc(cc2)C#N)CC1	Z1562120880
PP48e	ClCC(=O)N1CCCN(CC1)C(=O)Nc1ccccc1	Z567145816
PP48f	COc1ccc(cc1)S(=O)(=O)N1CCCN(CC1)C(=O)CCl	Z1562149684
PP48g	COc1ccc(cc1)C(=O)N1CCN(CC1)C(=O)CCl	Z561146200

Supplementary Table 2. Number of unique peptides detected across all experiments, before and after data filtering. The numbers quoted represent the number of peptides that pass each filtering step (1-5) when these filters are applied sequentially.

	**Initial screen: HEK293T lysate, 80 compounds**	**Initial screen: Jurkat lysate, 80 compounds**	**Validation: HEK293T lysate, 8 compounds**	**Lysate SAR: HEK293T lysate, 8 compounds**	**Live cell SAR: HEK293T live cells (2 h), 4 compounds**
Total samples	335	328	334	191	48
Total peptides	31107	29818	29096	27954	28664
1. Single DTB modification	30156	29034	28190	27198	28349
2. In ≥75% control samples	19037	18895	16123	15325	18175
3. Control sample CV <40%	15818	12200	11783	13215	16205
4. Most abundant/only cleavage product	14658	11231	10892	12174	15145
5. Present in ≥90% of all samples	13631	10321	9552	11174	14094

Supplementary Table 3. The diaPASEF method for chemoproteomics data acquisition on a Bruker timsTOF Pro 2. This diaPASEF method has been formatted to import into the acquisition software timsControl.

MS Type	Cycle Id	Start IM [1/K0]	End IM [1/K0]	Start Mass [m/z]	End Mass [m/z]	CE [eV]
MS1	0	-	-	-	-	-
PASEF	1	1.032	1.638	800	825	-
PASEF	1	0.835	1.031	600	625	-
PASEF	1	0.6	0.834	400	425	-
PASEF	2	1.056	1.638	825	850	-
PASEF	2	0.858	1.055	625	650	-
PASEF	2	0.6	0.858	425	450	-
PASEF	3	1.08	1.638	850	875	-
PASEF	3	0.884	1.079	650	675	-
PASEF	3	0.6	0.883	450	475	-
PASEF	4	1.106	1.638	875	900	-
PASEF	4	0.909	1.105	675	700	-
PASEF	4	0.6	0.908	475	500	-
PASEF	5	1.13	1.638	900	925	-
PASEF	5	0.934	1.129	700	725	-
PASEF	5	0.6	0.932	500	525	-
PASEF	6	1.154	1.638	925	950	-
PASEF	6	0.958	1.153	725	750	-
PASEF	6	0.6	0.957	525	550	-
PASEF	7	1.18	1.638	950	975	-
PASEF	7	0.983	1.179	750	775	-
PASEF	7	0.6	0.982	550	575	-
PASEF	8	1.204	1.638	975	1000	-
PASEF	8	1.006	1.202	775	800	-
PASEF	8	0.6	1.005	575	600	-

Supplementary Table 4. The diaPASEF method for global proteomics data acquisition on a Bruker timsTOF Pro 2. This diaPASEF method has been formatted to import into the acquisition software timsControl. This method has been optimised by pyDIAD.[10]

MS Type	Cycle Id	Start IM [1/K0]	End IM [1/K0]	Start Mass [m/z]	End Mass [m/z]	CE [eV]
MS1	0	-	-	-	-	-
PASEF	1	0.97	1.6	687.01	710.36	-
PASEF	1	0.82	0.97	538.27	554.96	-
PASEF	1	0.6	0.82	262.18	394.54	-
PASEF	2	0.99	1.6	710.36	736.37	-
PASEF	2	0.85	0.99	554.96	572.28	-
PASEF	2	0.6	0.85	394.54	424.74	-
PASEF	3	1	1.6	736.37	766.07	-
PASEF	3	0.86	1	572.28	589.61	-
PASEF	3	0.6	0.86	424.74	446.91	-
PASEF	4	1.01	1.6	766.07	800.93	-
PASEF	4	0.87	1.01	589.61	606.87	-
PASEF	4	0.6	0.87	446.91	466.76	-
PASEF	5	1.02	1.6	800.93	843.44	-
PASEF	5	0.88	1.02	606.87	625.79	-
PASEF	5	0.6	0.88	466.76	485.77	-
PASEF	6	1.03	1.6	843.44	898.45	-
PASEF	6	0.89	1.03	625.79	645.33	-
PASEF	6	0.6	0.89	485.77	503.79	-
PASEF	7	1.04	1.6	898.45	983.93	-
PASEF	7	0.9	1.04	645.33	665.38	-
PASEF	7	0.6	0.9	503.79	521.48	-
PASEF	8	1.09	1.6	983.93	1398.68	-
PASEF	8	0.93	1.09	665.38	687.01	-
PASEF	8	0.6	0.93	521.48	538.27	-

Supplementary References

1. Fedor. supervenn: precise and easy-to-read multiple sets visualization in Python. Preprint at https://doi.org/10.5281/zenodo.4016732.

2. White, M. E. H., Gil, J. & Tate, E. W. Proteome-wide structural analysis identifies warhead- and coverage-specific biases in cysteine-focused chemoproteomics. *Cell Chem Biol* **30**, 828–838.e4 (2023).

3. Douse, C. H. *et al.* TASOR is a pseudo-PARP that directs HUSH complex assembly and epigenetic transposon control. *Nat Commun* **11**, (2020).

4. Banerjee, S. *et al.* 2.3 Å resolution cryo-EM structure of human p97 and mechanism of allosteric inhibition. *Science (1979)* **351**, 871–875 (2016).

5. Wu, H. *et al.* Structural basis of allele variation of human thiopurine-S-methyltransferase. *Proteins: Structure, Function, and Bioinformatics* **67**, 198–208 (2007).

6. Jumper, J. *et al.* Highly accurate protein structure prediction with AlphaFold. *Nature* **596**, 583–589 (2021).

7. Keserű, G. M. *et al.* Design principles for fragment libraries: maximizing the value of learnings from pharma fragment-based drug discovery (FBDD) programs for use in academia. *J Med Chem* **59**, 8189–8206 (2016).

8. Wirth, M. & Sauer, W. H. B. Bioactive molecules: perfectly shaped for their target? *Mol Inform* **30**, 677–688 (2011).

9. Giordanetto, F., Jin, C., Willmore, L., Feher, M. & Shaw, D. E. Fragment Hits: What do They Look Like and How do They Bind? *J Med Chem* **62**, 3381–3394 (2019).

10. Skowronek, P. *et al.* Rapid and In-Depth Coverage of the (Phospho-)Proteome With Deep Libraries and Optimal Window Design for dia-PASEF. *Molecular & Cellular Proteomics* **21**, 100279 (2022).
