# Supplementary material for: Robust proteome profiling of cysteine-reactive fragments using label-free chemoproteomics
Source: Nat Commun. 2025 Jan 2;16:73. doi: 10.1038/s41467-024-55057-5 (PMC11697256; doi:10.1038/s41467-024-55057-5)
Supplement: Supplementary file 2 — Description of Additional Supplementary Information [file 41467_2024_55057_MOESM2_ESM.docx]

**Description of Additional Supplementary Files**

File Name: Supplementary Data 1

Description: All liganding events detected from screening 80 chloroacetamides at 50 µM in both HEK293T and Jurkat cell lysate.
