# Supplementary material for: Robust proteome profiling of cysteine-reactive fragments using label-free chemoproteomics
Source: Nat Commun. 2025 Jan 2;16:73. doi: 10.1038/s41467-024-55057-5 (PMC11697256; doi:10.1038/s41467-024-55057-5)
Supplement: Supplementary file 4 — Reporting Summary [file 41467_2024_55057_MOESM4_ESM.pdf]

nature portfolio

Corresponding author(s): Katrin Rittinger and Jacob Bush

Last updated by author(s): Nov 10, 2024

Reporting Summary

Nature Portfolio wishes to improve the reproducibility of the work that we publish. This form provides structure for consistency and transparency in reporting. For further information on Nature Portfolio policies, see our Editorial Policies and the Editorial Policy Checklist.

Statistics

For all statistical analyses, confirm that the following items are present in the figure legend, table legend, main text, or Methods section.

n/a	Confirmed	
☐	☒	The exact sample size (*n*) for each experimental group/condition, given as a discrete number and unit of measurement
☐	☒	A statement on whether measurements were taken from distinct samples or whether the same sample was measured repeatedly
☐	☒	The statistical test(s) used AND whether they are one- or two-sided<br>*Only common tests should be described solely by name; describe more complex techniques in the Methods section.*
☐	☒	A description of all covariates tested
☐	☒	A description of any assumptions or corrections, such as tests of normality and adjustment for multiple comparisons
☐	☒	A full description of the statistical parameters including central tendency (e.g. means) or other basic estimates (e.g. regression coefficient) AND variation (e.g. standard deviation) or associated estimates of uncertainty (e.g. confidence intervals)
☐	☒	For null hypothesis testing, the test statistic (e.g. *F*, *t*, *r*) with confidence intervals, effect sizes, degrees of freedom and *P* value noted<br>*Give P values as exact values whenever suitable.*
☒	☐	For Bayesian analysis, information on the choice of priors and Markov chain Monte Carlo settings
☒	☐	For hierarchical and complex designs, identification of the appropriate level for tests and full reporting of outcomes
☐	☒	Estimates of effect sizes (e.g. Cohen's *d*, Pearson's *r*), indicating how they were calculated

Our web collection on statistics for biologists contains articles on many of the points above.

Software and code

Policy information about availability of computer code

Data collection	All mass spectra were acquired using an Evosep One coupled to a Bruker timsTOF Pro 2 Mass Spectrometer, with the corresponding software provided by Bruker.
Data analysis	All chemoproteomics data was searched against an in-built library using Spectronaut (v16.0.220606.53000). All global proteomics data was searched via directDIA on Spectronaut (v18.6.231227.55695). Figures were generated either using Biorender (https://www.biorender.com/), PyMOL (v2.5.4), or Python (v3.8.18; with matplotlib v3.7.2 and seaborn v0.13.2).

For manuscripts utilizing custom algorithms or software that are central to the research but not yet described in published literature, software must be made available to editors and reviewers. We strongly encourage code deposition in a community repository (e.g. GitHub). See the Nature Portfolio guidelines for submitting code & software for further information.

Data

Policy information about availability of data

All manuscripts must include a data availability statement. This statement should provide the following information, where applicable:

- Accession codes, unique identifiers, or web links for publicly available datasets
- A description of any restrictions on data availability
- For clinical datasets or third party data, please ensure that the statement adheres to our policy

The proteomics data has been submitted to the ProteomeXchange Consortium via the PRIDE database under the identifiers: PXD054105, PXD054127, and PXD054145.

Research involving human participants, their data, or biological material

Policy information about studies with human participants or human data. See also policy information about sex, gender (identity/presentation), and sexual orientation and race, ethnicity and racism.

Reporting on sex and gender	N/A
Reporting on race, ethnicity, or other socially relevant groupings	N/A
Population characteristics	N/A
Recruitment	N/A
Ethics oversight	N/A

Note that full information on the approval of the study protocol must also be provided in the manuscript.

Field-specific reporting

Please select the one below that is the best fit for your research. If you are not sure, read the appropriate sections before making your selection.

☒ Life sciences ☐ Behavioural & social sciences ☐ Ecological, evolutionary & environmental sciences

For a reference copy of the document with all sections, see nature.com/documents/nr-reporting-summary-flat.pdf

Life sciences study design

All studies must disclose on these points even when the disclosure is negative.

Sample size	No statistical method was used to determine sample size. For lysate-based experiments, all compound-treated conditions were run in technical quadruplicate and at least 16 untreated (DMSO) samples were run. For cell-based experiments, all compound-treated conditions were run in biological triplicate and 12 untreated (DMSO) samples were run.
Data exclusions	No exclusion criteria were pre-established. Data quality of each spectra was determined by interpreting quality control criteria for individual runs. Peptides that were not associated with a human protein (according to UniProt) or that matched an entry in the contaminant database were removed from further analysis.
Replication	All data was acquired with technical (n=4) or biological replicates (n=3), and all observations are made in the context of any variability present in these replicates.
Randomization	Samples were randomised prior to LC-MS data acquisition.
Blinding	Blinding was not necessary as for the experiments described in the manuscript, as scientist bias could not have any impact on the data or the final measurement.

Reporting for specific materials, systems and methods

We require information from authors about some types of materials, experimental systems and methods used in many studies. Here, indicate whether each material, system or method listed is relevant to your study. If you are not sure if a list item applies to your research, read the appropriate section before selecting a response.

Materials & experimental systems

n/a	Involved in the study	
☒	☐	Antibodies
☐	☒	Eukaryotic cell lines
☒	☐	Palaeontology and archaeology
☒	☐	Animals and other organisms
☒	☐	Clinical data
☒	☐	Dual use research of concern
☒	☐	Plants

Methods

n/a	Involved in the study	
☒	☐	ChIP-seq
☒	☐	Flow cytometry
☒	☐	MRI-based neuroimaging

Eukaryotic cell lines

Policy information about cell lines and Sex and Gender in Research

Cell line source(s)	HEK293T (original origin: female human embyro) and Jurkat (original origin: male peripheral blood) cells were obtained from the Francis Crick Institute Cell Services STP.
Authentication	Cells were authenticated by STR profiling by Francis Crick Institute Cell Services STP.
Mycoplasma contamination	All cells were negative for mycoplasma contamination, based on fluorescence staining, agar culture, and PCR testing performed by Francis Crick Institute Cell Services STP.
Commonly misidentified lines (See ICLAC register)	No commonly misidentified lines were used.

Plants

Seed stocks	N/A
Novel plant genotypes	N/A
Authentication	N/A
